# The Effect of Void Arrangement on the Pattern Transformation of Porous Soft Solids under Biaxial Loading

**DOI:** 10.3390/ma14051205

**Published:** 2021-03-04

**Authors:** Hai Qiu, Ying Li, Tianfu Guo, Shan Tang, Zhaoqian Xie, Xu Guo

**Affiliations:** 1 State Key Laboratory of Structural Analysis for Industrial Equipment, International Research Center for Computational Mechanics, Department of Engineering Mechanics, Dalian University of Technology, Dalian 116024, China; hai_qiu@foxmail.com; 2 Department of Mechanical Engineering and Institute of Materials Science, University of Connecticut, Storrs, CT 06269, USA; Ying.3.li@uconn.edu; 3 Institute of High Performance Computing, A*STAR, Singapore 138632, Singapore; guotf@ihpc.a-star.edu.sg; 4 Ningbo Institute of Dalian University of Technology, No. 26 Yucai Road, Jiangbei District, Ningbo 315016, China

**Keywords:** porous soft solids, biaxial loading, void morphology, pattern transformation

## Abstract

Structural topology and loading condition have important influences on the mechanical behaviors of porous soft solids. The porous solids are usually set to be under uniaxial tension or compression. Only a few studies have considered the biaxial loads, especially the combined loads of tension and compression. In this study, porous soft solids with oblique and square lattices of circular voids under biaxial loadings were studied through integrated experiments and numerical simulations. For the soft solids with oblique lattices of circular voids, we found a new pattern transformation under biaxial compression, which has alternating elliptic voids with an inclined angle. This kind of pattern transformation is rarely reported under uniaxial compression. Introducing tensile deformation in one direction can hamper this kind of pattern transformation under biaxial loading. For the soft solids with square lattices of voids, the number of voids cannot change their deformation behaviors qualitatively, but quantitatively. In general, our present results demonstrate that void morphology and biaxial loading can be harnessed to tune the pattern transformations of porous soft solids under large deformation. This discovery offers a new avenue for designing the void morphology of soft solids for controlling their deformation patterns under a specific biaxial stress-state.

## 1. Introduction

Cellular or porous solids (e.g., honeycombs and foams made from metals and polymers), have been extensively used in practical engineering. Linking the macroscopic properties with the microstructure of voids has been studied in Gibson’s [1] book. It continues to be a hot topic, due to the emergence of mechanical metamaterials [2,3,4,5] and architectured materials [6,7,8]. On one hand, porous solids can be used to design the light-weight structures to meet the specified requirements (e.g., high energy absorption or high stiffness/strength to weight). In order to achieve these requirements, the constituent materials are usually elastoplastic or elasto-viscoplastic (e.g., polycarbonate, copper and polyester urethane). The microstructure of voids is not required to be periodic or evenly distributed. For instance, when the cellular foams or polymer honeycombs fabricated by polyester urethane or polycarbonate are made under uniaxial or biaxial compression, the buckling of cellular wall will form the local deformation bands. The mechanical behaviors of porous materials begin at the approximately linear elastic stage, terminate at the ultimate load, and are followed by a wide range of load plateaus [9,10,11,12,13,14]. In this way, high energy absorption will be realized. An analogous phenomenon is also found for metals (e.g., nickel-based superalloy [15] and copper [16]).

On the other hand, homogeneous and reversible pattern transformation under specific loading conditions (e.g., usually compression) can be harnessed to realize specific functions or attributes. Examples were demonstrated in the experiments of [17] and finite element analysis on the unit cell by [18]. They observed a kind of pattern transformation with alternating orthogonal elliptic voids under uniaxial compression through the porous polymers. This pattern transformation is caused by the elastic instability, such as the buckling of beam-like ligaments, in cellular solids. The pattern transformation is homogeneous without the localized deformation bands found in cellular solids with more randomly distributed voids [1]. In their applications, these materials usually should have a quick response to the external stimulation and return to the original shape after unloading. These materials are usually hyperelastic, such as silicone rubber [19,20,21], polydimethylsiloxane (PDMS) [22,23] or photoelastic elastomer [24,25]. Pattern transformation resulting from the instability opens up a new method for the manufacturing of soft matters with adjustable acoustic, optical and electrical properties [26,27,28,29,30,31].

When designing porous materials with high stiffness, strength, energy absorption or pattern transformation, void morphology and loading conditions play important roles. Porous auxetic lattice structures have been used as the reinforcements in the soft material matrices recently, which can achieve significant improvements on the stiffness and energy absorption [32]. The triangular lattice is used to replace the cell wall of the honeycombs to form a hierarchical configuration, which can lead to shape integrity and high energy dissipation with any large deformation [33]. Pore shape’s effect on the pattern transformation in cellular elastic materials has been investigated by [28]. Most of the studies mentioned above only consider the deformation behavior of porous materials under uniaxial compression. [10,11] designed a new experimental apparatus to perform biaxial crushing of polycarbonate. Their creative device can accurately apply biaxial compression for crushing; nevertheless, their device cannot apply compression and tension at the same time, as the platen cannot grasp the specimen [34,35]. Qiu et al. have designed an experimental device to apply biaxial loading [21]. In their work, specimens with a 15×15 square array of circular voids were studied under different biaxial loading ratios. A new pattern transformation under combined compression and tension has been identified.

In this work, we continued our previous work and focused on the effects of void arrangement and distribution on the pattern transformation of cellular silicone rubber under biaxial loading by integrating experiments and simulations. Both oblique and square lattices of circular voids were considered. We tried to understand whether pattern transformation can occur under different void morphologies and biaxial loadings, particularly when one direction is under compression and the other is under tension. We organized this paper into the following parts. An introduction to the four different types of porous silicone rubber, the experimental apparatus and method, preparation of the specimens and the numerical calculation method are all in Section 2. With the constitutive model of determined material parameters, the buckling and post-buckling responses of cellular silicone rubber for biaxial loading are further studied through experimental measurements and computer simulations. Section 3 provides the numerical examples and corresponding experimental results. Finally, conclusions are presented in Section 4.

## 2. Experiment and Numerical Simulation

### 2.1. Experimental Method

**Figure 1:**
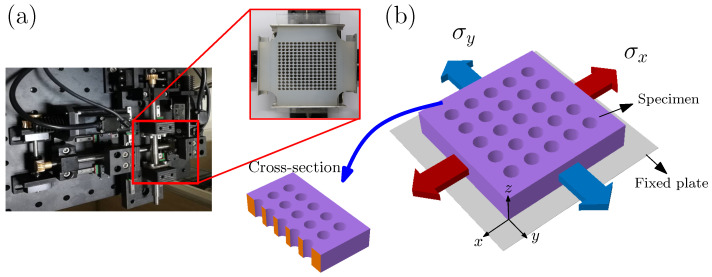
(**a**) A self-designed biaxial loading apparatus for porous solids. (**b**) Schematic of porous solids under biaxial loading. The upper and lower surfaces of the samples were closely fixed by two thin sheets (made of PMMA). Here, only the thin sheet of the lower surface is drawn.

Before the experiment, a biaxial loading apparatus was designed, which can realize the biaxial loading process with different proportions (see Figure 1a). The general arrangement of the experimental apparatus and the specimen is shown in the red box of Figure 1a. Figure 1b shows the schematic of porous solids under biaxial loading, and a three-dimensional cross-section view. The upper and lower surfaces of the sample were closely fixed by two thin-plates made of polymethyl methacrylate (PMMA) to avoid buckling of the surface. Of course, the thin-plate must be transparent to record the deformation information of the voids during the loading process. The washing-liquid was also applied to eliminate the friction between the thin-plate and sample.

A biaxiality ratio is introduced for the experiments, which can be defined as:

(1) $$\gamma =\frac{\varepsilon_{y}}{\varepsilon_{x}}$$

where $\varepsilon_{x}=\delta_{x}/L_{x}$ and $\varepsilon_{y}=\delta_{y}/L_{y}$. $L_{x}$ and $L_{y}$ are the original length of the sample in the direction of *x* and *y*, respectively. $\left\{\delta_{x},\delta_{y}\right\}$ denote the displacement in *x* and *y* loading direction. To eliminate the potential viscoelastic effect of porous solids, the strain rate should be set very small and approximately $1\times 10^{-3}$/s. Then the experiment is conducted through parameters $\left\{L_{x},L_{y},\gamma \right\}$. It should be noted that there will be a special loading state (uniaxial compression) in the later part of this study. For the convenience of description, γ=0 is introduced, which is different from the definition in Equation (1). The tensile and compressive strain of the specimen are realized by the designed clamps, and the applied strain can be obtained by measuring the length of the specimen in the loading process.

### 2.2. Preparation of Porous Samples of Silicone Rubber

**Figure 2:**
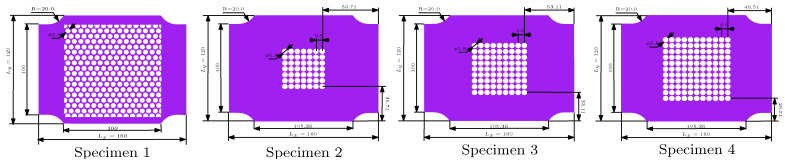
Geometry and dimensions of four specimens used in the present study. Specimen 1 had an oblique lattice of voids, while specimens 2–4 had square lattices of voids. The void diameter and spacing were the same for these specimens (mm).

Figure 2 shows four types of specimens for biaxial loading, following the typical design principle [36,37]:

- Specimen 1, comprising a microstructure of a 15×15 oblique array of circular voids. All the voids have identical size with diameter 5.8 mm. They are arranged with 6.6 mm center-to-center spacing vertically and horizontally.
- Specimens 2–4, comprising a microstructure of a 7×7, 9×9, 11×11 square arrays of circular voids. All the voids have identical size with diameter 5.8 mm. They are arranged with 6.6 mm center-to-center spacing vertically and horizontally.

For specimens 1–4, their dimensions are marked in Figure 2, and the thickness of all specimens was 10 mm.

In order to fabricate the specimen as shown in Figure 2, we first use 3D printing technology and epoxy materials to print the corresponding mold. Then the silicone rubbers, the raw material of porous samples, need to be prepared. The silicone rubber used in this paper is made by mixing A (vinyl with hydrogen group) and B (vinyl silicone with organic platinum catalyst) liquids in a ratio of 1:1. By controlling the content of A, silicone rubber with different Young’s modulus and hardness can be manufactured. The fully stirred mixture is placed in the vacuum tank to remove the gas. Then the mixture is poured into a mold after removing the gas. In order to accelerate the solidification, we put the mold in the incubator and keep it at 50 ℃ for two hours. To separate the sample from the mold more easily, the mold surface is sprayed with releasing agent.

### 2.3. Material Models for Silicone Rubber

The deformed behavior of silicone rubber can be described by hyperelastic model. F=∂x/∂X is the deformation gradient mapping a material point from the reference position X to its current location x. The Odgen model implemented in ABAQUS [38] is adopted. The free energy density takes [39]:

(2) $$W=\frac{2\mu }{\alpha }\left({{\bar{\lambda }}_{1}}^{\alpha }+{{\bar{\lambda }}_{2}}^{\alpha }+{{\bar{\lambda }}_{3}}^{\alpha }-3\right)+K_{m}{\left(J-1\right)}^{2}$$

where $\lambda_{i}\left(i=1,2,3\right)$ are the stretches in the principal direction. ${\bar{\lambda }}_{i}$ are the deviatoric part of $\lambda_{i}$, which can be obtained by ${\bar{\lambda }}_{i}=J^{-1/3}\lambda_{i}$ and J=detF. α is a material constant. Elastic modulus *E* and Poisson’s ratio ν have the classical relationship between μ and $K_{m}$. The different Poisson’s ratios can be obtained by adjusting the ratio between $K_{m}$ and μ. Finally, the first Piola–Kirchhoff (PK) stress can be written as [40]:

(3) $$P_{ij}=\frac{\partial W}{\partial F_{ij}}$$

In previous work [41], the Neo–Hookean model was often chosen to describe the mechanical properties of silicone rubber. However, our previous work [21] shows that it could not accurately simulate the biaxial compression/tension behavior of silicone rubber. Therefore, the Odgen model is adopted to strengthen the predictive ability of the model. It is shown evidently in our previous work that the Neo–Hookean model can well describe the mechanical response of materials in compression, but it fails in tension [21]. By fitting the experimental results of silicone rubber, the parameters μ and α of Odgen’s model are 0.12 and 4.9, respectively. The elastic modulus and Poisson’s ratio of silicone rubber are 0.375 MPa and 0.499, respectively, based on the measured stress–strain response shown in our previous work [21].

### 2.4. Buckling and Post-Buckling Analysis

Generally, to simulate the pattern transformation of the cellular solids, two-step finite element analyses are carried out: buckling and post-buckling analysis. Buckling analysis of finite size regions was studied by previous works [42,43,44,45,46,47] and summarized in detail in ABAQUS theory manual [38]. An imperfection of the eigenmode obtained by buckling modes from linear instability analysis is introduced into FE mesh. Then, the mesh is scaled by a factor ω and perturbed by the corresponding eigenmode. The pattern of porous solids may be sensitive to imperfections. However, our simulation results show that the results are almost the same for different imperfections. Thus, the factor of imperfection *W* is set to 0.00034R, where *R* represents the radius of the void.

## 3. Results and Discussion

**Figure 3:**
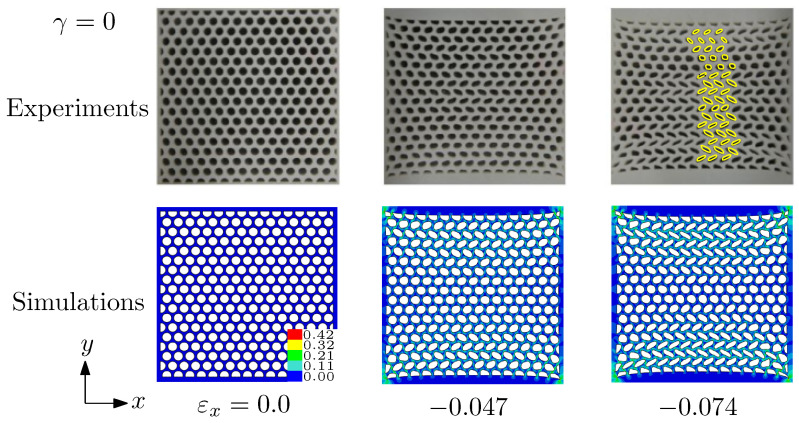
Deformed morphologies of specimen 1 at different applied strains (0%, −0.047 and −0.074, respectively) for biaxiality ratio γ=0 (uniaxial compression) predicted by the simulations and the experiments. The color bar shows the effective strain of the simulations.

Figure 3 shows the deformed morphologies of specimen 1 (see Figure 2) at different levels of the applied strain $\varepsilon_{x}$. $\varepsilon_{x}=0.0$ represents the undeformed configurations. After the initial uniform deformation, the chevron pattern begins to appear at a critical strain around 0.047, and then the chevron pattern aggravates with the increase of compressive strain. This chevron pattern of voids is highlighted by the yellow lines, which results from the elastic instability demonstrated by the bucking analysis. This pattern transformation is almost the same as that in our previous work [21] under the combined tension and compression. We name it pattern transformation II, which is similar to the Figure 30 of [24]. The deformation of the specimen is not localized into a row or diagonal band for the elastic instability under the uniaxial compression. Uniaxial compression on porous soft solids with an oblique square lattice of voids (7 by 7) has been studied by [24]. Here we demonstrate that we can reproduce their results by specimen 1. Note that specimen 1 with circular holes is different from the sample with square holes used in [24] under uniaxial compression.

**Figure 4:**
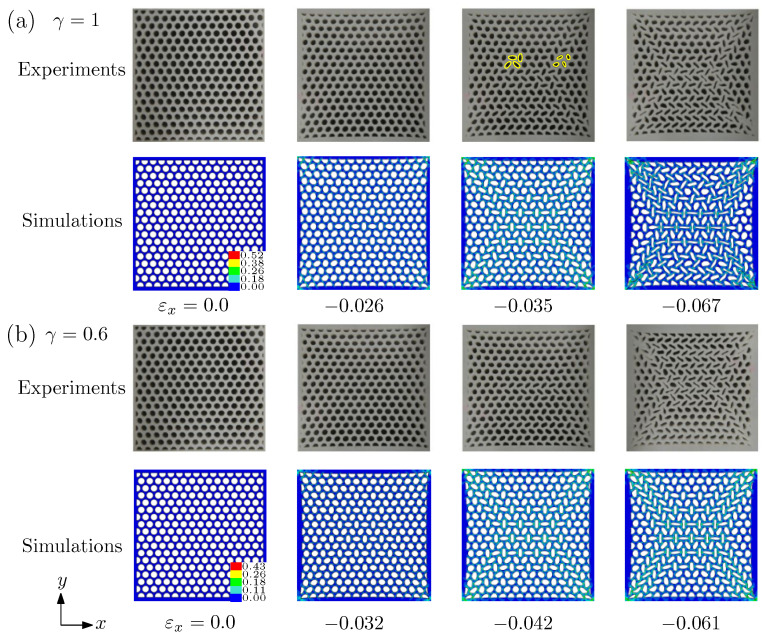
Deformed morphologies of specimen 1 at different applied strains predicted by the simulations and the experiments. The color bar shows the effective strain of the simulations. (**a**) γ=1; (**b**) γ=0.6.

We then consider two compressive cases, where sample 1 is compressed along *x* and *y* planes with γ=1 and 0.6 in Figure 4a,b respectively. The deformed morphologies under different levels of the strain obtained by the simulations and experiments are also presented. Let us first explain the case of γ=1. The results of γ=0.6 are similar to those of γ=1. Under the condition of equibiaxial compression γ=1, it can be found that the central part of the sample forms a pattern with alternating elliptic voids, which is different from that shown in Figure 3 of uniaxial compression γ=0. This case clearly illustrates the importance of biaxial loadings. We also found that the elliptic voids are not orthogonal but have an inclined angle (see Figure 4a), as highlighted by yellow outlines. For the convenience of description, it is called "pattern transformation III" (PT III). The critical strain for γ=1, where PT III takes place, is lower than that of γ=0.6. The critical strain is 0.026 for γ=1 while 0.032 for γ=0.6. The buckling analysis further affirmed that the pattern transformation is the result of elastic instability events, although it is not shown directly here. With the increase of the applied strain, the prolate voids become more prolate and the oblate ones become more oblate. By comparing the results of experiments and simulations shown in Figure 4, it can be known that the numerical results are consistent with the experimental results.

**Figure 5:**
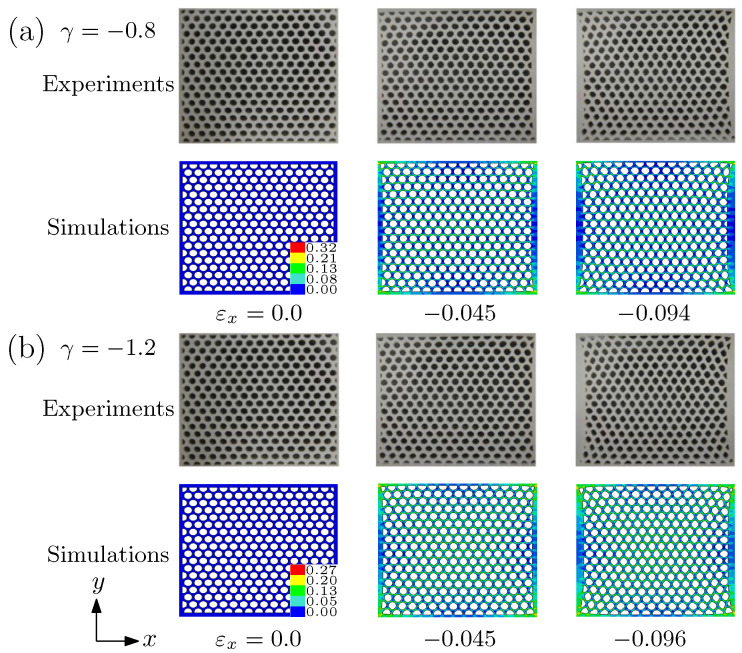
Deformed morphologies of specimen 1 at different applied strains predicted by the simulations and the experiments. (**a**) γ=−0.8; (**b**) γ=−1.2. The tensile loading along *y* direction is imposed.

The deformed morphologies at different levels of applied strain of specimen 1 for γ=−0.8 and −1.2 are given in Figure 5. The deformation of voids is almost homogeneous. Even the compression imposed along *x* direction was large, the pattern transformation was not still observed, although the circular shape of voids became elliptical. It is interesting that the large tension in *y* direction can inhibit PT III. Thus, the biaxiality ratio and oblique lattice of circular voids will offer a new avenue for controlling the deformation modes of porous materials.

**Figure 6:**
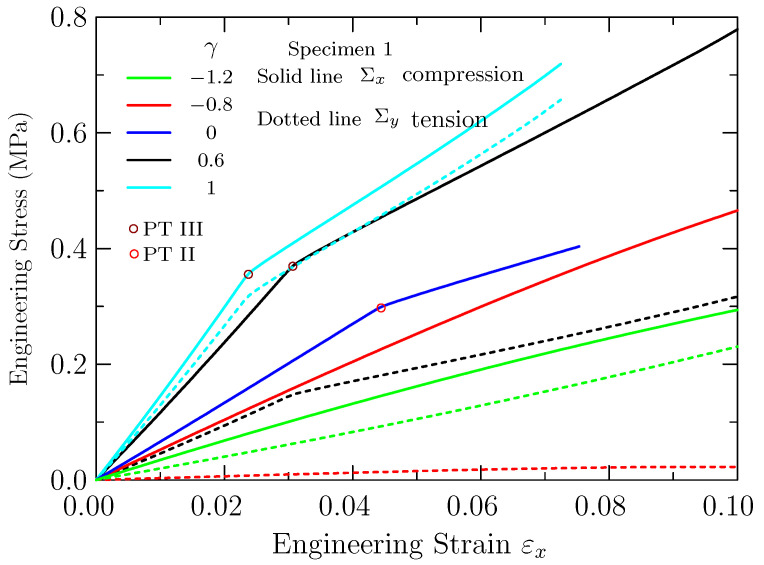
The engineering stress–strain responses for specimen 1 calculated at different biaxiality ratios. The critical points for the onset of "pattern transformation III" (PT III) or PT II are marked by dark-red or red circles on these curves. Pattern transformation III was only observed for specimen 1 with an oblique lattice of voids under biaxial compression. The solid lines and the dotted lines stand for the engineering stress $\Sigma_{x}$ in the direction of *x* and $\Sigma_{y}$ in the direction of *y*, respectively.

Figure 6 shows the engineering stress–strain responses for specimen 1 calculated at different biaxiality ratios. The solid lines and the dotted lines in the figure stand for the engineering stress $\Sigma_{x}$ in *x* direction and $\Sigma_{y}$ in *y* direction. Each color represents a biaxiality ratio. Note that $\Sigma_{y}$ is zero when uniaxial compression γ=0 is applied. The critical points for onset of PT III for different biaxiality ratios are marked on these curves by unfilled circles. From Figure 6, we can see that $\Sigma_{x}$ increases linearly with increase of the applied strain at beginning. When the PT III occurs, the engineering stress $\Sigma_{x}$ increases continuously but the slope of curves declines. Differently from the results given in [10,11,24], the compressive stress does not attain a plateau and shows a hyperelastic behavior. The possible reason is that the silicone rubber involved in present work is different from what they used (polycarbonate and photoelastic elastomer PSM-4). The silicone rubber can be purchased directly. The similar results can be obtained with the other soft solids behaving hyperelastically, such as polydimethylsiloxane (PDMS). When the biaxiality ratios are negative (e.g., γ=−0.8 and −1.2), PT III cannot be observed. Hence, there is no transition point for the slopes of these stress–strain curves.

We then studied the effect of void number on the deformation pattern of porous soft solids. Note that in specimens 2–4, the sizes of the initial circular void and void spacing were the same. It is shown in our previous work that the pattern transformation I (PT I) always occurs under biaxial compression or uniaxial compression. The definition of pattern transformation I is given in our previous paper [21]. It is also shown in the next paragraph. We focus on the combined tensile and compressive loading in this work.

**Figure 7:**
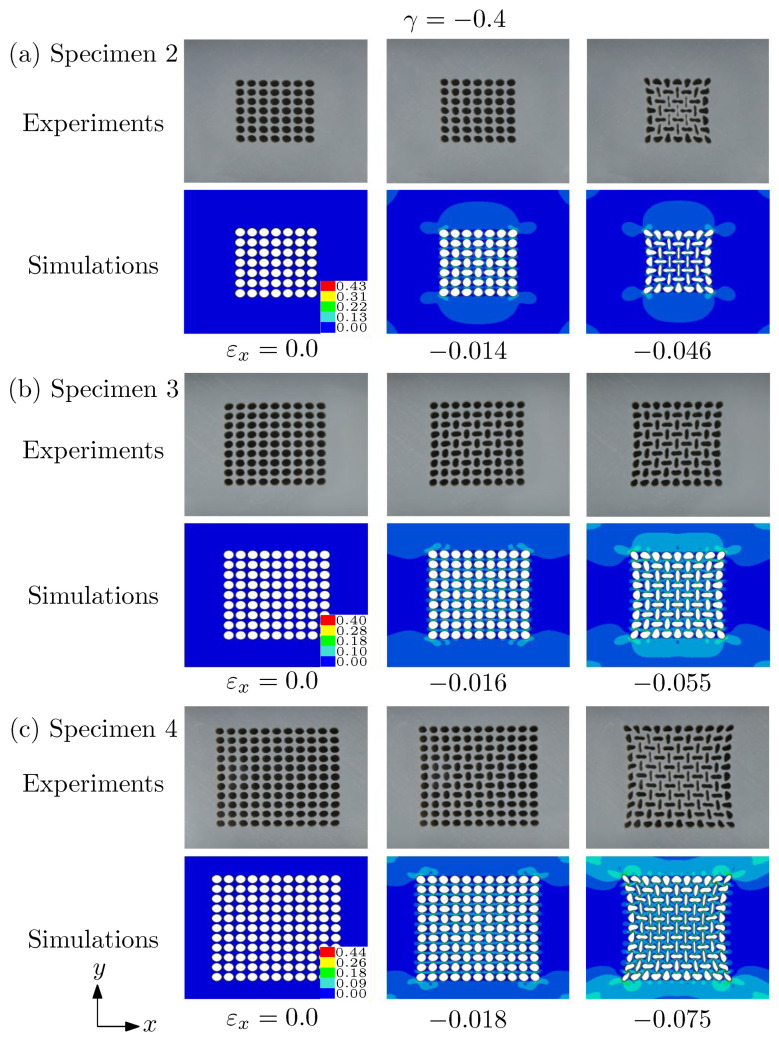
Deformed morphologies at different applied strains predicted by the simulations and the experiments. (**a**) Specimen 2; (**b**) specimen 3; (**c**) specimen 4. Under this biaxiality ratio γ=−0.4, only PT I (alternating orthogonal elliptic voids) was discovered. The color bar shows the effective strain of the simulations.

We first show the results of γ=−0.4. The deformed morphologies at different applied strain obtained by the simulations and experiments are shown in Figure 7. Generally, even the number of voids for three different specimens is different, and the observed deformation process is almost the same. At first, the voids undergo a homogeneous deformation. At a critical strain, the relatively affine-like deformation goes into a structure with alternating orthogonal ellipses, called pattern transformation I (PT I) in our previous work [21]. This is the only observed pattern transformation under uniaxial compression, given in [17,18,24]. The second column of Figure 7 demonstrates the moment when the PT I occurs. With an increase of applied strain, the deformation of voids is accentuated. That is, the prolate pores become more prolate and the oblate ones become more oblate. Although the trend of deformation is almost the same, we can see that the critical point for the onset of PT I increases as the number of voids increases.

**Figure 8:**
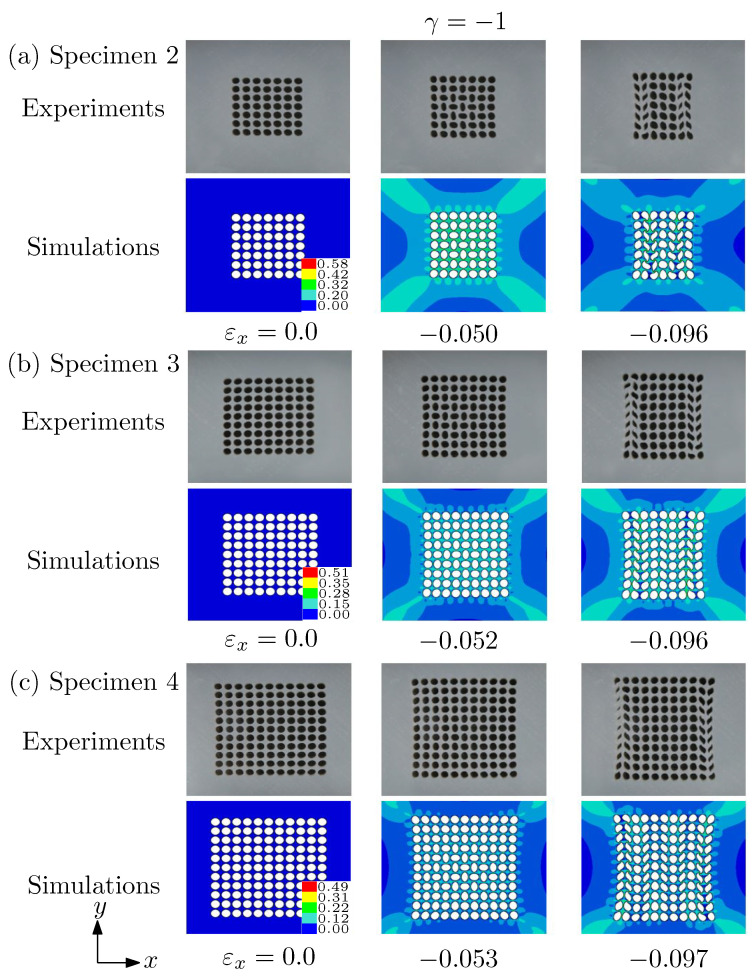
Deformed morphologies at different applied strains predicted by the simulations and the experiments. (**a**) Specimen 2; (**b**) specimen 3; (**c**) specimen 4. Under this biaxiality ratio γ=−1, both pattern transformation I (alternating orthogonal elliptic voids) and pattern transformation II (chevron voids) could be observed. The color bar shows the effective strain of the simulations.

Figure 8 also shows the deformation configurations at different strains obtained by the simulations and experiments for γ=−1.0. We can see that the porous solids experience two different pattern transformations. First, the relatively affine-like deformation goes into a pattern with alternating and orthogonal ellipses at a critical strain and PT I occurs. Second, with the imposed strain further increasing, the pattern of voids observed for spacemen 1 under uniaxial loading appears, defined as pattern transformation II (PT II) in our previous work [21]. The second column of Figure 8 shows the moment when the PT I occurs, while the third column for the onset of PT II. Comparing with that given in Figure 7 for γ=−0.4, we can observe that the critical strains for the onset of PT I and II both increase with the number of voids increasing. Interestingly, the PT II is more clear in numerical computations than that in experiments.

Although there are some differences between the simulations and experiments, the results obtained by the biaxiality ratio (−1 and −0.4) can offer a quick avenue to control the pattern transformation of cellular solids, and design tunable acoustic, optical and electric apparatuses by using the PT I. Let us explain the idea based on specimen 2 with a 7 by 7 square lattice of voids. Under the loading mode of γ=−1, when the strain in *x* direction is about 0.05, the transition mode PT I appears. However, with a little more tension in the direction of *y*, the pattern of PT I will disappear. This state of change only needs a little more tensile strain in the direction of *y*, and the change process is very fast.

**Figure 9:**
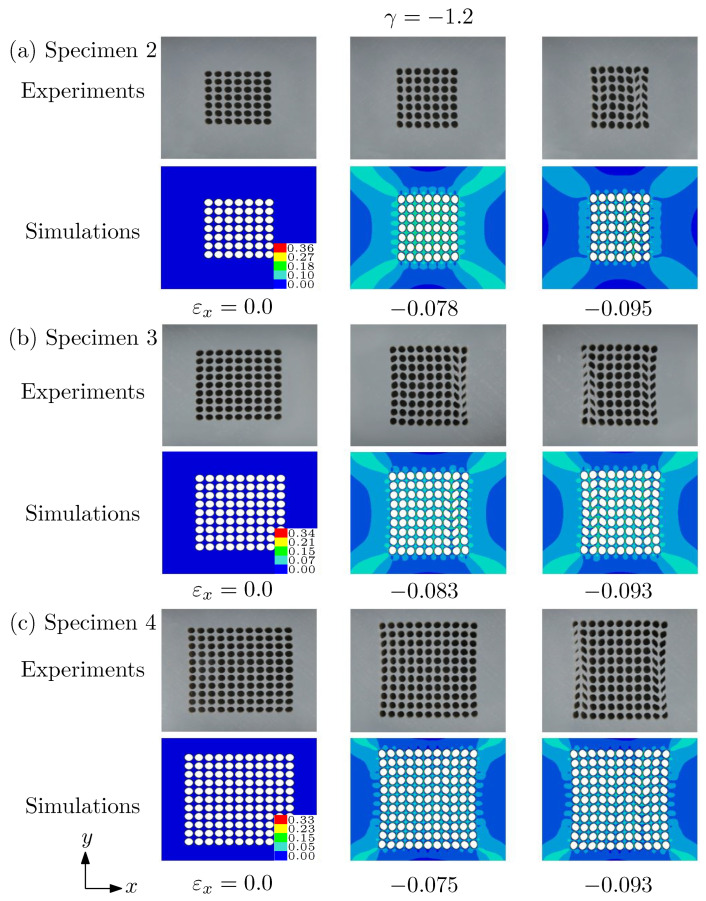
Deformed morphologies at different applied strains predicted by the simulations and the experiments. (**a**) Specimen 2; (**b**) specimen 3; (**c**) specimen 4. Under this biaxiality ratio γ=−1.2, only pattern transformation II (chevron voids) could be observed. The color bar shows the effective strain of the simulations.

With the increase of the compressive strain in the direction of *x*, the tensile strain in the *y* direction increases simultaneously. If the imposed strain in the direction of *y* is larger than that in the direction of *x*, then, e.g., γ=−1.2. It can be seen from the Figure 9 that PT I cannot occur but PT II can.

**Figure 10:**
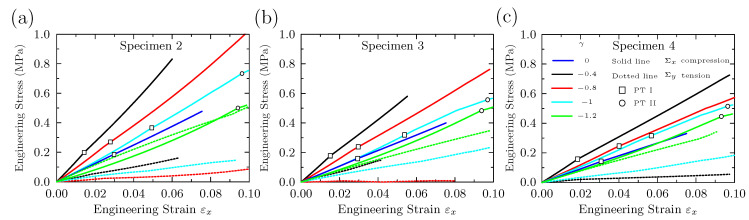
The engineering stress–strain responses for (**a**) specimen 2, (**b**) specimen 3 and (**c**) specimen 4 calculated at different biaxiality ratios. The critical strains of PT I and PT II are marked by unfilled squares and circles on these curves. The solid lines and the dotted lines stand for the engineering stress $\Sigma_{x}$ in the direction of *x* and $\Sigma_{y}$ in the direction of *y*, respectively.

The engineering stress–strain responses for specimens 2, 3 and 4 calculated at different biaxiality ratios are shown in Figure 10a–c respectively. The solid lines and the dashed lines in the figure stand for the engineering stress $\Sigma_{x}$ in *x* direction and $\Sigma_{y}$ in *y* direction. Each color represents a biaxiality ratio. The critical points for onset of the PT I or PT II for different biaxiality ratios are marked on these curves by unfilled symbols of squares and circles. The $\Sigma_{x}$ increases linearly with the increase of the applied strain at the beginning. When the PT I occurs, the engineering stress $\Sigma_{x}$ increases continuously but the slope of curves declines. With the increase of the imposed strain, the $\Sigma_{x}$ increases linearly. When the PT II takes place, the slope of curves slightly declines and the compressive stress increases continuously. With the biaxiality ratios γ increasing (e.g., γ=−1,−1.2), the PT II can also occur, which is marked with the unfilled circle on the curves. After the appearance of PT II, the slope of the curves decreases further. Among specimens 2–4, the volume fraction of voids in specimen 2 was the lowest, resulting in the largest stress response.

## 4. Conclusions

In this work, the pattern transformations of porous silicone rubber for different void morphologies and biaxial loadings were studied. Crisscross specimens with 15×15 oblique lattices of circular voids and 7×7, 9×9 and 11×11 square lattices of circular voids were considered. An extensive range of biaxiality ratios from 1 to −1.2 was used. The numerical simulations of the crisscross specimen under biaxial loading were simulated by the finite element method. Our results demonstrate that voids’ arrangement and biaxial loading condition have great impacts on the deformation behavior of porous soft solids, especially pattern transformation. Three pattern transformations have been found in the present study. The main conclusions are as follows:

1. The different pattern transformations (e.g., alternating orthogonal ellipses PT I and chevron pattern PT II) of the porous soft solids with square lattices of voids can be controlled quantitatively by adjusting number of voids and biaxiality ratios through experiments and simulations. This discovery provides a new way to design the void morphologies of soft solids for controlling their deformation patterns under a specific biaxial stress-state.
2. A new type of specimen with oblique lattices of circular voids was designed. In the experiment, a new pattern transformation (the elliptic voids were not orthogonal but with an inclined angle PT III) was observed that has rarely been reported in previous studies. This discovery offers new opportunities for fabricating tunable apparatuses and imprinting complex patterns of soft materials.

## Data Availability

Not applicable.
